# Summer pollen flora in rural and urban central England dominated by nettle, ryegrass and other pollen missed by the national aerobiological network

**DOI:** 10.1007/s10453-022-09759-2

**Published:** 2022-10-11

**Authors:** Mary Hanson, Geoff Petch, Thor-Bjørn Ottosen, Carsten Skjøth

**Affiliations:** 1 School of Science and the Environment, University of Worcester, Henwick Grove, Worcester, WR2 6AJ UK; 2 Department of Environmental Science - Atmospheric Measurements, Aarhus University, Frederiksborgvej 399 building 7413, 4000 Roskilde, Denmark; 3 Department of Air and Sensor Technology, Danish Technological Institute, Kongsvang Allé 29, 8000 Aarhus C, Denmark

**Keywords:** eDNA, Cyclones, Air, Microbiology, Biostatistics

## Abstract

Abundance and diversity of airborne pollen are important to human health and biodiversity. The UK operational network collects airborne pollen from 8 flowering trees, grasses and three weeds using Hirst traps and microscopic identification from urban areas. Knowledge of total pollen diversity and differences between rural and urban zones is limited. We collect environmental DNA (eDNA) from air during summer and autumn over 3 years with mini cyclones from one urban and one rural site. Data are analysed using next generation sequencing and metabarcoding. We find the most common genus, *Urtica* (57%), is also identified by the national network. The grasses *Lolium* (10%), *Agrostis* (2%) and *Holcus* (1%) are in the national network grouped at family level, while *Brassica* (2%), *Chenopodium* (1%), *Impatiens* (2%), *Plantago* (4%) and *Tilia* (7%) are not part of the UK operational network. DNA from 138 genera was identified, where 2% of the sample could not be associated with specific genera. 40% of the sample was classified better using eDNA methods at the genus level, than by optical methods. We calculate Bray–Curtis dissimilarity for the rural and urban zones and find a systematic difference in biodiversity. Overall, this shows airborne DNA reveals more information than methods based on morphological differences. The results also suggest data from sites located in large urban areas will be less representative for less populated rural areas. This presents a dilemma in balancing a network and the associated costs delivering health relevant information to the most populated areas vs. a nation-wide approach.

## Introduction

Airborne pollen is involved in many different aspects covering health, environment and climate (Hornick et al., 2022). For decades mature networks have collected airborne pollen in many countries, most often with the focus on improving life for hay fever sufferers (Ziello et al., 2012). Analysing long-term data sets is vital and often results in pollen calendars for either single stations or national networks (Adams-Groom et al., 2020) based on internationally accepted standards for recognition (Galán et al., 2014) and analysis and terminology (Galán et al., 2017). Traditional methods focus on limited numbers of species. Accuracy assessments are usually to a single or few species, genera or families. The methods are based on capture of the airborne pollen grains and analysing the morphological characteristics under a microscope. This approach often recognises a limited number of species at either the genus or family level. In the UK this is limited to pollen from eight trees and three weeds at the genus level and grasses at the family level (Adams-Groom et al., 2020; McInnes et al., 2017). A greater number of taxa are recorded through abundant monitoring stations across Europe depending on the local flora (e.g. *Pinus*, *Olea, Cupressaceae* and Amaranthaceae) (Buters et al., 2018; Ziello et al., 2012); however, the combined number of taxa recorded in the European network, remains much lower compared to what is expected from next generation sequencing methods (de Vere et al., 2012). New approaches based on collection with cyclones, next generation sequencing and metabarcoding have shown to provide much more taxonomical detail (Brennan et al., 2019; Hanson et al., 2022) but these approaches are still so new, that it is unclear how much more detail it is possible to obtain. The purpose of this study is to analyse data from an urban and nearby rural site with next generation sequencing over a 10 week period over three consecutive years covering the period when grasses and weeds are flowering to test whether there is a significant difference between the airborne pollen spectrum at the genus level and to explore whether the molecular approach provides substantially more information about species diversity than the optical microscopy approach used in the operational network.

## Methods

Airborne material was collected with two Burkard multi-vial cyclones in Worcestershire, UK; one rural site (52.2544°, − 2.2537°) and one urban (52.1969°, − 2.2422°). Material was collected for 10 weeks from the beginning of July during the years 2016–2018 (Hanson et al., 2022), hence providing 420 daily samples. DNA was extracted according to (Hanson et al., (2022) and pooled into 6 annual aggregations for each of the years 2016–2018. Samples were profiled using the ITS2 region and Illumina MiSEQ sequencing was performed externally by Eurofins Genomics. Bioinformatic analysis was performed in R with taxonomic assignment against the UNITE eukaryotic database v8.3 and using Dada2, phyloseq and vegan packages as detailed in Hanson et al. (2022) and postprocessing of the Amplicon Sequence Variant (ASV) data for pollen producing plants (Anthophyta and Coniferophyta) was done by c-shell scripting. The most prevalent pollen producing plants at each site were determined at the genus level by examining the top ten taxa and total biodiversity was calculated by enumerating all possible taxa at the genus level. ASV data were used to calculate alpha and beta diversities in a similar way as for spores in the study by Hanson et al. (2022). For beta diversity we use nonmetric multidimensional scaling (NMDS) based on Bray–Curtis dissimilarity to study how different the rural and urban sites are in terms of pollen flora. Shannon and Simpson (1-D) alpha-diversity indices were used to examine overall pollen diversity and evenness Simpsons’ diversity index is weighed towards species abundance, while the Shannon index is the most frequently used diversity measure as it also accounts for information entropy, the uncertainty of correctly predicting which species an individual will belong too. A t test was applied to plant ASV data agglomerated at genus taxonomical level to examine whether there was a statistically significant difference in the number of plant genera at each site.

## Results

**Fig. 1:**
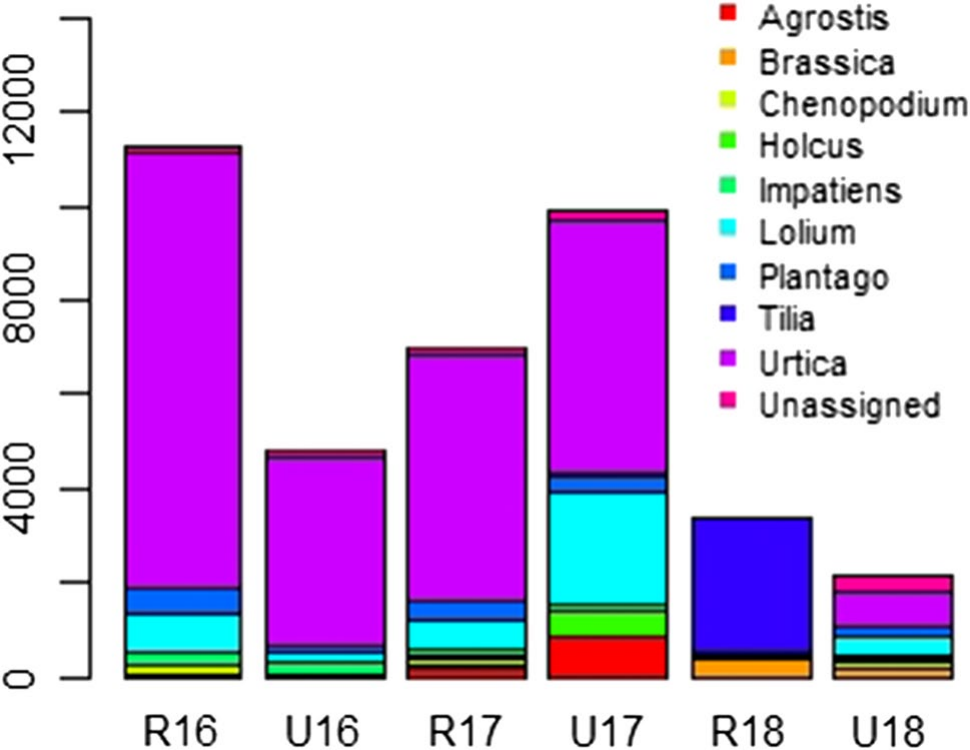
Amplicon Sequence Variant (ASV) numbers of the most frequently observed plant genera observed during the years 2016–2018, separated as annual values for either the rural or urban location

The total taxonomic assignments (ASVs) for pollen producing plants are shown in Table 1. Genus level assignment produces the most taxonomic classifications as several taxa cannot be classified at species level. The 10 most common species include the genera *Urtica* (57%); the grasses of *Lolium* (10%), *Agrostis* (2%) and *Holcus* (1%); *Tilia* (7%); *Plantago* (4%); *Impatiens* (2%); *Brassica* (2%) and *Chenopodium* (1%) (Fig. 1).

**Table 1:** Number of taxa (ASVs) at different taxonomic levels and Simpson and Shannon diversity indices calculated from the entire sample at the urban and rural sites during each of the years 2016, 2017 and 2018

	2016		2017		2018	
	Rural	Urban	Rural	Urban	Rural	Urban
No. Taxa (ASVs)						
Total taxa	50	29	34	51	28	45
Taxa assigned to Species	28	18	22	31	13	24
Taxa assigned to Genus	41	22	26	36	16	33
Taxa assigned to Family	21	17	16	20	14	23
Diversity index						
Simpson	0.41	0.45	0.49	0.71	0.39	0.89
Shannon	1.16	1.18	1.33	1.89	0.95	2.74

**Fig. 2:**
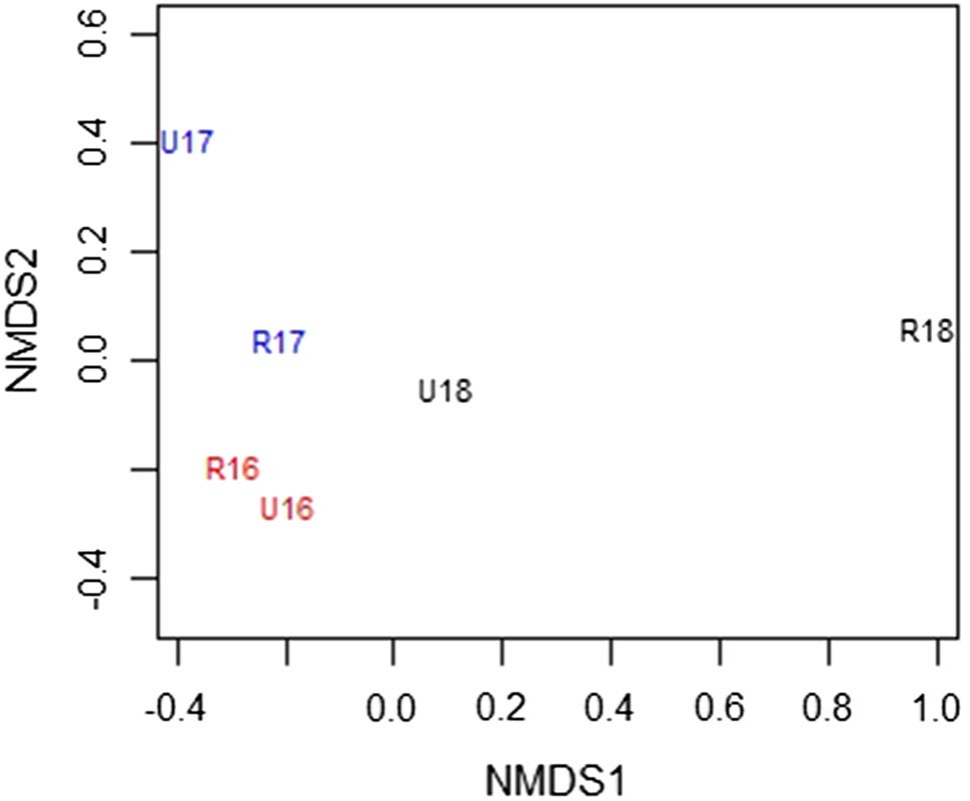
Nonmetric multidimensional scaling (NMDS) based on Bray–Curtis dissimilarity using all genera found at the rural (R) and urban (U) sites over the period 2016–2018

Only 2% of the sequences could not be assigned to a genus. There is no significant difference in the total number of genera at each site (*t* = − 0.411, df = 3.54, *p* = 0.7); however, for most genera there is more than tenfold variation in ASV number between years. In 2017 the difference between rural and urban zone is less than 20% for the genera *Plantago* and *Urtica*. In all other cases the difference is much larger and often a factor of 3 to 5. The Bray–Curtis dissimilarity indices covering the 10 most frequent species for each of the three years 2016–2018 are 0.41, 0.21 and 0.89, respectively, and 0.28 for the 3 years combined. NMDS based on Bray–Curtis dissimilarity (Fig. 2) shows that the rural site in 2016 and 2017 was similar while 2018 was quite different.

It also shows that the urban site was quite dissimilar across all years. Table 1 shows that the Simpson indices vary from less than 0.4 to almost 0.9, while the Shannon indices vary from near 1.0 to more than 2. The year 2018 had both the highest (urban) and lowest (rural) values of both indices indicating that this year showed a large difference in diversity between sites. The high Shannon index of 2.74 at the urban site indicates there may be many evenly distributed species present, while the rural site in the same year shows lower species richness and evenness, suggesting possible dominance of one or more species. The Simpsons’ diversity index, which is weighed towards species abundance, also showed greater evenness within species at the urban site in 2018 and lowest at the rural site for the same year.

## Discussion

Here, we consistently find that during the summer–autumn period substantially more information can be revealed using molecular methods, thereby complementing traditional pollen counting. The new information can contribute to better taxonomical information regarding grasses (Poaceae) for example, with *Lolium*, *Agrostis* and *Holcus* distinguished, or by the detection of genera not monitored as part of the national aerobiological network (e.g. *Brassica*, *Plantago* or *Impatiens*). Molecular methods could supplement routine monitoring for these genera which include important allergenic species, such as *P. lanceolata* (Sousa et al., 2014) and respondents to climate change, such as *Impatiens* (Rewicz et al., 2022). Furthermore, the risk of misidentification is very small using molecular methods; a previous study for the UK (de Vere et al., 2012) showed that the accuracy for correct taxonomical identification at the genus level is near 100%, but somewhat lower at the species level. A limitation of molecular methods is the possibility a species may not be assigned, for example we observed no species resolution of *Quercus* or *Artemesia* and therefore microscopic counts for these genera are likely more cost-effective for monitoring within the national network. Additionally, molecular profiling is not fully quantitative as variation in relative abundance may result from multiple permutations of changes in individual taxa. To overcome this the inclusion of known concentrations of standards are required, which is an emerging area of research.

The dissimilarity indices suggest that for 2 out of the 3 years there was a large similarity between the rural and urban sites which are located about 7 km apart. In the third year there were large differences and a detailed investigation of the numbers revealed that these differences were found among a larger number of prevalent species. A previous study on grass pollen using cyclones and metabarcoding (Brennan et al., 2019) found large variations between the appearance and abundance of grass pollen from different genera. This study covered 1 year focusing on the summer and urban locations. This rural–urban transect study extends that previous study by showing large year-to-year variations as well as rural–urban variations. It has also been shown for the UK area that local emission sources can make important contributions to pollen or spore levels (Apangu et al., 2020; Frisk et al., 2022). This may explain how a few genera appear related to the more dissimilar years. This suggests that a nationwide ecological assessment of the airborne pollen flora, ideally, should cover several years as well as the rural–urban transect. A similar conclusion on the rural–urban transect was recently found for fungal spores (Hanson et al., 2022) and the observed changes were directly connected to climate change, similar to what has been observed for airborne pollen (Ziello et al., 2012). However, most observational sites are located in urban zones (Buters et al., 2018) and very few in rural regions. The results from this study covering the rural–urban transect complemented by the study of Hanson et al., (2022) therefore questions whether the scientific community has fully detected the impact from climate change on airborne pollen and spores. The detection approach using cyclones and methods from molecular genetics does not offer the high temporal resolution seen from the operational networks or newer real-time devices (Crouzy et al., 2016). Instead, it offers higher possibility for taxonomical identification and a cost-effective approach for long-term assessments, when compared with either labour intensive pollen counting or real-time instruments with high capital costs.
